# Inclusive Redistribution and Perceptions of Membership: A Cross-National Comparison

**DOI:** 10.1177/00104140251342924

**Published:** 2025-05-27

**Authors:** Allison Harell, Keith Banting, Will Kymlicka

**Affiliations:** 1 381704 Université du Québec à Montréal, Montréal, QC, Canada; 2 4257 Queen’s University, Kingston, ON, Canada

**Keywords:** membership, immigration, welfare state, deservingness, national identity

## Abstract

Immigrants tend to be seen as less deserving of welfare benefits than native-born citizens, but little consensus exists to explain this finding or how to build greater public support for more inclusive policies. Recent work suggests that support for redistribution may be tied to citizens’ perceptions of the “membership commitment” of immigrants. This study provides the first systematic test of this hypothesis in the comparative setting using an original seven country survey conducted in 2021–2022. The survey explored perceptions of immigrants’ membership commitment in the host society in seven liberal democracies and their effect on public support for the extension of social benefits to immigrants. The study shows that immigrants systematically suffer a “membership penalty” within host societies across a wide range of states with different citizenship and welfare regimes, with important consequences for welfare state support.

## Introduction

The impact of immigration and ethnic diversity on social solidarity in democratic societies continues to be a contested topic of both political and scholarly debate. Central to these debates has been concern that immigration and diversity weaken the welfare state and the redistributive role of government. This concern is a long-standing one. More than three decades ago, Gary Freeman (1986) warned that large-scale migration would lead to the ‘Americanization’ of European welfare states. This anxiety expanded in the early decades of this century. Redistribution, analysts reasoned, rests on the foundation of a shared sense of community, and growing ethnic, racial, and religious diversity seemed likely to weaken the bonds that tied co-nationals together, eroding public support for collective projects, including the welfare state. The intensity of this original concern has faded in recent years, in part because the welfare state has obviously not collapsed in the face of contemporary immigration. However, a second version of the debate has taken centre stage. This version holds that although citizens of contemporary democracies remain committed to their major social programs, the inclusion of newcomers in social benefits is politically explosive. In this version, the underlying sense of a shared community continues to sustain the welfare state, but it also establishes sharp boundaries that define who is included and who is excluded from social protection, leading to a form of “welfare chauvinism”. The welfare state supports members of the historic majority, protecting “us,” rather than newcomers, who are not seen as part of “us.” In this view, a redistributive state may remain a feasible political project, but an *inclusive* redistributive state is not.

This study seeks to contribute to our understanding of the factors that influence the inclusion or exclusion of immigrants in welfare state regimes. We argue that support for redistribution generally, especially inclusive forms of redistribution, is powerfully tied to perceptions of immigrants’ “membership commitment”: that is, whether the native-born see immigrants as committed to the larger society and willing to make sacrifices for it. Such commitment to one’s country of residence presents a novel reconceptualization of how citizens judge immigrants’ inclusion within the political community. We find that across seven liberal democracies, immigrants suffer a “membership penalty” – they are seen as less committed to the larger society – and these membership perceptions have a powerful impact on public support for the inclusion of immigrants in the benefits of the redistributive state. Moreover, these membership effects are not reducible to other factors that affect support for such redistribution, such as racial prejudice, or perceptions of whether immigrants are hardworking, or the native-born population’s sense of national identity. Although the membership penalties vary across countries with different citizenship and welfare regimes, they have a powerful impact on support for inclusive redistribution in all of our cases.

## Immigration, Diversity, and the Welfare State

In the early 2000s, the fear that diversity was eroding interpersonal trust, solidarity and redistribution received widespread scholarly attention (Alesina & Glaeser, 2004; Putnam, 2007). A small industry quickly arose trying to test the impact of immigration and diversity on support for the welfare state, but the empirical analyses generated contradictory findings. Although some studies found evidence that immigration reduces public support for social programs or dampens social spending (Eger, 2010; Soroka et al., 2016), others do not (Crepaz, 2008; Finseraas, 2011; Gerdes, 2011; van Oorschot & Uunk, 2007). Indeed, a survey of 464 articles found that “there are nearly as many studies rejecting the negative effects of diversity as arguing for them” (Schaeffer, 2014: 4; also Van der Meer & Tolsma, 2014). Another review added that the effects, whether positive or negative, seem to be small (Stichnoth & Van der Straeten, 2013; see also Portes & Vickstrom, 2011). Clearly, a tension between diversity and solidarity is not a universal reality. Although racial diversity has played into anti-welfare politics in some countries, including the United States, the welfare state has also continued to display the considerable political durability identified in earlier research (Pierson, 1994).

Although ethnic and racial diversity may not inevitably erode the welfare state, the inclusion of immigrants in its benefits is widely contested, a phenomenon that has been christened ‘welfare chauvinism’ (Koning, 2019; Sainsbury, 2012). Aided by an innovative index of welfare exclusion, recent scholarship has illuminated the general pattern of welfare exclusion across democratic countries (Koning, 2021, 2022). Three themes stand out from this research. First, welfare chauvinism is not a new phenomenon. Indeed, exclusion of immigrants from social benefits has declined across western democracies compared with the 1980s. Second, the level of exclusion varies significantly across types of programs. Access to social assistance is most controversial, and many countries have restricted access to this benefit over time, dramatically in some cases.

Finally, and most relevant in this context, there is dramatic and persistent variation in the levels of welfare exclusion across countries (Koning, 2022). This variation defies easy explanation. One obvious possibility is that the level of exclusion is influenced by the nature of the welfare state regime itself, with exclusion more marked in liberal welfare states than in social democratic ones. However, this explanation cannot easily explain the differences within regime types. For example, among social-democratic welfare states, exclusion is low in Norway but high in Denmark; among liberal-democratic regimes, exclusion is low in Canada but high in the United States.

In this context, focusing on public attitudes offers a way forward. Studies have measured public conceptions of the deservingness of different groups for social benefits and have found that immigrants rank at the bottom of the deservingness hierarchy everywhere in Europe and beyond (van Oorschot, 2006, see also Kootstra, 2017; Magni, 2022; Voss et al., 2020). The strength and steepness of the hierarchies in public attitudes differ across countries, but the tendency to exclude seems ubiquitous.

Why are immigrants seen as less deserving of social benefits? Past research tends to focus on two broad types of explanatory factors. The first set of factors focus on outgroup antipathy grounded in perceptions of cultural distance and cultural threat. Immigrants are seen as a culturally distant outgroup, and xenophobia and other forms of outgroup antipathy drive down support for redistribution simply because the other group is “other” and this othering comes with a host of negative stereotypes. Racial attitudes have long been linked to less support for welfare benefits, both in the US (Gilens, 2009) and comparatively (Ford, 2016; Harell et al., 2016). This is in part because those in need of social benefits are stereotyped as failing to demonstrate key cultural norms and behaviours (Katz, 1989). This perception activates group stereotypes that categorize (black and brown) immigrants as lazy, dishonest, violent, criminal or other familiar stereotypes, pushing down support for public benefits. Indeed, immigrants’ country of origin is strongly related to anti-immigrant attitudes more generally (Alarian & Neureiter, 2021).

The idea that the cultural threat of immigrants drives down support for redistribution is supported in multiple studies that document lower support for benefits to immigrants from less culturally proximate countries (Ford, 2016; Kootstra, 2016; Reeskens & van der Meer, 2019). By contrast, when immigrants share a salient ethnic identity with the native-born population, the penalty for immigrants is less steep, and can even disappear (Kootstra, 2016).

The second set of explanatory factors focus on perceptions of immigrants’ economic impact, and whether immigrants are likely to generate economic benefits or economic costs. In some contexts, immigrants are viewed as an economic threat, either because they are assumed to take more out of welfare state programs than they contribute, or because they compete for specific jobs or public benefits (such as public housing). Previous research suggests that publics largely prefer higher educated and more skilled immigrants, regardless of whether their country of origin is seen as culturally close or culturally distant (Hainmueller & Hopkins, 2015; Valentino et al., 2019). When it comes to welfare benefits, an individuals’ work ethic and their efforts to find employment have long been used as markers of deservingness (Goulding & Middleton, 1982; Katz, 1989). More recent work on immigrants’ deservingness suggests that previous employment history and efforts to find a job increase public support for immigrants’ access to social benefits, although whether work history and work effort is sufficient for immigrants to be seen as equally deserving remains contested. Kootstra (2016), for example, shows that ethnic minorities in the second generation are seen as equally deserving when they have demonstrated “good” behavior, such as a track record of working and efforts to find a new job. By contrast, Magni (2022) finds that previous work history only reduces, but does not eliminate, the immigrant-native gap in deservingness, and Reeskens and van der Meer (2019) conclude that looking for work does *not* close the immigrant-native deservingness gap.

### Outgroup Bias versus Ingroup Inclusion: The Role of Membership Commitment

Clearly, there is evidence that welfare chauvinism is tied to perceptions of both cultural and economic threat. However, the existing literature is focused primarily on explaining the drivers of outgroup exclusion and has devoted less attention to explaining the sources of ingroup inclusion. In our view, inclusion into a solidaristic community is not simply a matter of overcoming perceptions of outgroup threat, bias and prejudice, but also involves active processes of membership-making, and of inclusion into a “we”.

The wider literature provides important hints about possible pathways to such inclusion. One such hint comes from studies of the role of legal status in the political community and a second hint comes from the literature on national identity. For some analysts, inclusion depends on attaining formal membership in the political community through the legal status of citizenship. Indeed, there is some evidence that once immigrants acquire citizenship, they are more likely to be accepted as deserving members (Levy & Wright, 2020; Sobolewska et al., 2017). However, as we see below, there is also evidence that holding citizenship does not guarantee that members of minority groups will be seen as deserving of inclusive redistribution. Goodman (2014) similarly concludes that formal acquisition of citizenship in contemporary liberal democracies is rarely viewed by the public or by policy-makers as sufficient. Instead, she argues that “civic integration defines membership and belonging in the contemporary nation-state” (2014, p. 16). States increasingly expect immigrants to display evidence of ‘civic integration’ and promote (or compel) civic integration through courses and tests that seek to instill knowledge of the national language, knowledge of the society’s history and culture, knowledge of its laws and institutions, job training, civic volunteering, political participation, and so on. The growing popularity of such civic integration policies might suggest that the public too has similar expectations of immigrants.

However, immigrants’ participation in civic integration programs also does not ensure that the majority population sees them as deserving of social benefits. Integration programs involve a heterogeneous and constantly shifting mix of cultural, social, political and economic dimensions. As many critics have argued, this has meant that goalposts of “integration” are constantly changing in ways that make it difficult for immigrants to demonstrate they meet the expectations of the state or of society generally (Entzinger, 2006). Not surprisingly, Alarian and Neureiter (2021) suggest that civic integration policies do not seem to lead to more positive attitudes toward immigrants among citizens, either in general or specifically in relation to support for immigrants’ inclusion within core redistributive schemes of the state.^1^

A second hint about pathways to inclusive solidarity, which is more helpful in the current context, comes from the literature on national identity. According to Miller (1995), for example, the shared society that is the locus of attachment and commitment is (and must be) the “nation”, and so attachment and commitment can be operationalized in terms of “national identity”. In effect, then, Miller predicts that those with a stronger sense of national identity will have a stronger sense of redistributive solidarity (Miller, 2017, p. 73; Miller & Ali, 2014; Miller, 2006, p. 332). Attempts to test the national identity argument have had mixed results, as Miller himself admits (Miller & Ali, 2014). More recent authors have attempted to nuance the hypothesis by distinguishing national “identity”, “attachment”, “belonging”, and “pride”, and have argued that each of these has a distinctive relationship to solidarity, although even with these refinements, the results remain inconclusive (e.g., Gustavsson & Stendahl, 2020; Rapp, 2022; Bruinsma & Mußotter, 2023).

We would argue that while the national identity argument is correct to focus on the idea of affective commitment to the larger society, it starts from the wrong end of the telescope. It focuses exclusively on the survey respondents’ own identity and attachment, whereas what matters for an ethics of membership is people’s perceptions of *others’* attachment. My willingness to redistribute to you depends, not just on how attached I am to the larger society, but on whether I think you are attached to the larger society.^2^ If individuals who belong to a particular subgroup are seen as being unwilling to commit to the larger society, they are likely to be seen as less deserving of the distinctive benefits associated with the welfare state. Insofar as the welfare state rests on an ethic of membership, then membership perceptions should be politically consequential. The case of welfare chauvinism is arguably a perfect illustration of this phenomenon. Defenders of welfare chauvinism often have a strong sense of attachment to the nation, but they do not think immigrants have the right sort of attachment and therefore exclude them from the obligations and benefits that come from this attachment.

Some defenders of the national identity argument respond to this worry by distinguishing “ethnic” and “civic” forms of national identity and asserting that people who endorse a civic conception of the nation would be more likely to endorse more inclusive forms of redistribution. However, this still starts from the wrong end of the telescope. The fact that a respondent endorses a civic conception of the nation tells us who they see as *eligible* to join the shared society built upon an ethics of membership: they are open to the possibility that racial and religious minorities can become members in good standing who are committed and attached to the larger society. In that sense, embracing a civic conception of the nation may be a necessary condition for supporting inclusive redistribution. But the crucial question remains: do respondents think that immigrants and other minorities *in fact* exhibit this commitment and attachment, or do they view certain minorities as indifferent to the larger society, or even actively disloyal to it?^3^ And here the evidence is clear: those who endorse a civic conception of national identity can be as discriminatory towards immigrants – particularly Muslims – as defenders of ethnic nationalism (Simonsen & Bonikowski, 2020; Turgeon et al., 2019). The fact that someone endorses a civic conception of national identity does not tell us, by itself, whether they think that immigrants in their countries comply or not with the ethics of membership.

In short, while there are now many sophisticated and nuanced versions of the “national identity argument”, we believe that perceptions of the level of commitment of immigrants to their new political community is both a theoretically and empirically rich lens through which to study whether immigrants are judged to be deserving of redistribution.

Our hypothesis is that inclusion into solidarity is fundamentally a matter of *perceived* commitment. We predict that the willingness of majorities to support redistribution to immigrants depends in part on whether immigrants are seen as committed to the larger society. Of course, this immediately raises the question of what it means to be committed to the larger society. One dimension of commitment might indeed be the willingness to make an economic contribution, as emphasized by the existing literature on economic factors. But commitment, in our view, is much broader than that. Membership in a new political community, we argue, is perceived to be in part a give and take with others in society, but also an affective commitment to do one’s part to make sure the political community thrives and that all members are taken care of. To use old-fashioned language, our hypothesis is that majorities will be more willing to support redistribution if they see immigrants and other minorities as “loyal” and “patriotic”. We believe that these perceptions of membership commitment – what we will call “membership perceptions” in short – are central to public attitudes towards redistribution.

We also believe that these are not reducible to a simple economic exchange, nor are they necessarily related to prejudice directly. An immigrant group may be seen as committed to society even if they are poor or a racial minority or lack the formal status of citizenship; and conversely, a group may be seen as indifferent to the wider society even if they are white, well-off, and have formal citizenship. Of course, here as elsewhere, negative attitudes tend to run together and feed off each other, and so we expect various grounds of “undeservingness” to be correlated. But our hypothesis is that membership perceptions play a significant role, even when these other factors are controlled for, because they capture the extent to which immigrants are seen as fulfilling expectations of being a committed member of the in-group, the political community they now call home.

### Evidence of Membership Penalties

The idea that perceptions of commitment play an important role in shaping attitudes towards immigrants is noted in recent social psychology literature (e.g., Jasinskaja-Lahti et al., 2020; Kunst et al., 2019), but is surprisingly absent in the recent welfare chauvinism literature, with a few exceptions. Recent work in Sweden by Sandelind and Hjerm (2022) find that when Swedes view immigrants (or at least non-European immigrants) as more “emotionally attached” to the nation, they are more supportive of both general and inclusive forms of redistribution. Previous work in Canada has shown that membership perceptions more generally are indeed an important driver of redistributive solidarity, not captured by or reducible to the standard explanations (Banting et al., 2020; Harell, Banting, Kymlicka, & Wallace, 2022).^4^ Drawing on a custom-designed survey of 2100 respondents in Canada in 2017, a new battery of questions was designed to test the majority’s perception of the membership commitment of three minorities in Canada - immigrants, Indigenous peoples and French-speaking Quebecers - and its relations to redistribution toward these minorities. The findings showed that:

- all three minorities are seen by others in society as less committed to the larger society;
- this perceived lack of commitment has a powerful effect on support for inclusive redistribution: the less these minorities were seen as committed to Canada, the less respondents supported redistribution to them; and
- these “membership penalties” remain significant even when we control for standard measures of outgroup affect; standard measures of laziness/control; and the respondents’ strength of national identity.

Are these results generalizable and robust? Is this unique to Canada or Sweden, or do we see a similar pattern across other Western democracies? To help answer that question, we have administered a survey in seven countries in North America and Europe.^5^ We focus our comparative study on immigrants.^6^ Immigrants are not only a group that can be compared across countries, but also are a group that faces significant – though varied - barriers to inclusion within the welfare state in our case countries. Our paper has two objectives:

(a) First, we seek to assess whether immigrants in all countries are seen as less committed or attached to the larger society. In other words, are immigrants, on average, viewed as less than fully committed to their host political community. The size of these differentials is likely to vary across countries for a variety of reasons. For example, differentials in perceived membership commitment might be smaller in countries with more liberal naturalization and multiculturalism policies, both of which signal confidence in immigrants’ membership commitment. However, we expect to find “membership penalties” for immigrants in all countries.
(b) Second, we ask whether membership perceptions shape support for inclusive redistribution in all countries. Again, the relative impact of membership perceptions compared to other factors is likely to vary. In some countries, perceptions of membership commitment might strongly affect support for redistribution; in other countries, it might have a weaker effect. For example, membership perceptions might play a smaller role in shaping overall redistributive support in countries with more universalistic welfare states, which tend to dampen “deservingness” judgements. However, here again, we expect to find a positive relationship between membership perceptions and inclusive redistribution across all countries.

## Data and Methods

To explore these questions, we draw on the Intergroup Relations and Foundations of Solidarity (IRFS) survey (*n* = 13,760) conducted in seven countries in North America and Europe from Sept. 2021 to July 2022 (Harell, Banting, Kymlicka, Larsen, et al., 2022). The countries include Canada (*n* = 2046), US (*n* = 2020), Great Britain (*n* = 2006), Italy (2033), France (*n* = 1898), Sweden (*n* = 1884) and Denmark (*n* = 1863). Surveys were collected by Cint, an online opt-in panel provider, where we used representative quotas for age, gender, region and education (with an additional language quota in Canada).^7^ Opt-in online panels are a cost-effective and efficient non-probabilistic sampling technique that have become increasing common methods for survey collection (Olson et al., 2020). In general, opt-in panels often capture the direction and relationship of variables but tend to be less reliable in estimating the overall population levels (Dassonneville et al., 2020; Fournier et al., 2015).

The data here are analysed in a pooled model with country controls, as well as individually by country. The 20-minute survey was fielded in the country’s official language(s) to respondents 18 years and older who were citizens or permanent residents at the time of the survey. All data is weighted, though weighting had little effect on estimates or relationships.^8^

The seven countries included in this survev vary in terms of welfare state design. Canada, the US and Great Britain are liberal welfare states; Denmark and Sweden are social democratic regimes; France and Italy are continental regimes.^9^ These countries also vary in the nature and size of their immigrant communities, the origins of immigrants and history of immigration. Canada and the US are settler societies which were colonized by European settlers, and both have significant Indigenous communities as well as historic minorities (particularly Black Americans and Canadians, and a significant French minority in Canada). The European case countries vary in the levels of ethnic and racial diversity present, linked both to their histories of colonization, as well as more recent immigrant flows. The types of immigrant flows vary as well because of the nature of the immigration system in place, a country’s geography, among other reasons. These seven countries therefore represent a large range of democratic systems in which to examine the nature of membership perceptions and their impact on attitudes toward redistribution.

Our core outcome of interest is attitudes toward redistribution. In this context, we distinguish between two forms of redistribution: general redistribution and inclusive redistribution. General redistribution is measured using a three-item scale using standard questions about the redistribution of wealth and the welfare state. These include responses on a 5-point agree/disagree scale for: 1) The government should provide social assistance for those in need; 2) The government should see to it that everyone has a decent standard of living; and 3) Government should redistribute income from the better-off to those who are less well off.^10^ The scale is standardized to run from 0 to 1.

We are particularly interested, however, in the second form of redistribution. The issue here is how well immigrants are integrated into the core welfare state programs in the country. As the welfare chauvinism literature makes clear, citizens can want a robust welfare system but also prefer to exclude immigrants from those benefits. As a contrast to this, we measure what we call inclusive redistribution. We measure this through an additive scale composed of three agree/disagree questions that broadly mirror the general redistribution items above. Specifically, the questions ask respondents to think about immigrants in the country and whether it is the government’s responsibility to: 1) ensure immigrants have access to social assistance when they need it; 2) provide a decent standard of living for immigrants in (country); and 3) reduce income differences between immigrants and other (nationals).^11^ The scale runs from 0 to 1, with higher scores indicating greater support for redistribution to immigrants. Note that in models estimating inclusive redistribution, we always include a control for general redistribution. This is because we are interested in isolating support for immigrants’ inclusion in welfare schemes separately from overall support for the welfare state. This is particularly important because past research suggests that support for the welfare state can remain high in the face of immigration because people may become more exclusive in who they want to access such benefits (Reeskens & van Oorschot, 2012).

We are interested in how perceptions of membership influence attitudes toward redistribution, and to capture these attitudes, we develop a series of six questions that tap the extent to which residents in a country see immigrants as committed to a national “we”.^12^ Are they seen as caring about the others in the society as we would expect among members of a political community? Are they seen as thinking of themselves as members of that community who are willing to contribute to it? Specifically, respondents in each country were asked to compare immigrants to “other (nationals)” on a five-point scale from much less to much more where the midpoint implies that they are similar to other nationals. The items included how much immigrants 1) identify with the country; 2) care about the concerns and needs of other (nationals); 3) are willing to make sacrifices for others in society; 4) contributing their fair share by working and paying taxes; 5) how proud they are to be (nationals); 6) how willing immigrants would be to volunteer to fight for the country if it was involved in a war.^13^ These items allow for an exploration of the extent to which non-immigrants view immigrants in the country as committed to the political community. The final scale runs from 0 to 1 and achieves a high level of internal reliability (Cronbach’s alpha = .80 in pooled sample, and >.71 in each individual country). Note that .5 is a key point on this scale indicating that immigrants are viewed as similar to nationals in the country in their level of membership commitment. Scores above .5 suggest that immigrants are viewed as *more* committed than other nationals, whereas scores below .5 indicate immigrants are viewed as *less* committed.

As we expect both attitudes toward redistribution and perceptions of membership to be correlated with other sociodemographic and attitudinal variables, we include a host of control variables in multivariate models. In terms of sociodemographic controls, we include gender, income, education, foreign-born, age and religion. We also control for a wide variety of attitudinal measures that provide alternative explanations of attitudes toward redistribution. These include:

- Respondents’ own attitudes toward the nation (i.e., the strength of their national identity, and its civic/ethnic content);
- Perceptions of immigrants’ control/responsibility for their disadvantage (i.e., are immigrants seen as lazy and hence responsible for their fate);
- Inter-group Affect (i.e., dislike of immigrants as measured by a feeling thermometer)^14^

Finally, we also control for ideological orientation of the respondent, using a three-category variable for left-leaning, centrist, and right-leaning, respondents.^15^ Detailed coding of each variable is available in Appendix A1. These models thus include several of the standard factors that have been used to explain attitudes toward redistribution in the literature and allow us to test the extent to which membership attitudes provide a unique, additional explanation for both general and inclusive redistributive attitudes.^16^

## Analysis

**Figure 1.**
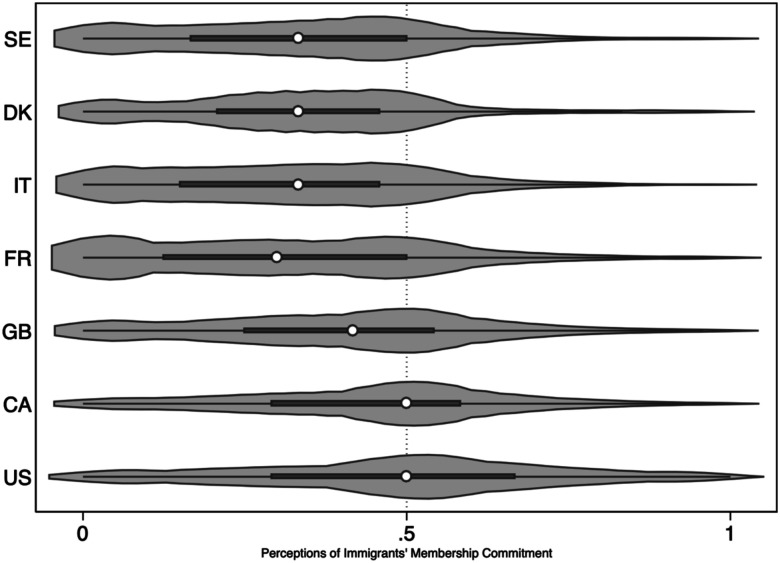
Membership Perceptions across Countries. Note: Violin plot by country, excluding foreign-born. Weighted.

The first step in our analysis is to assess whether immigrants in fact face a membership penalty across the countries in this study: are they seen as less committed to the larger society? Figure 1 presents a violin plot of the membership score by country.^17^ What is striking immediately is that in every country, scores are skewed toward the negative end of the scale. While the median score is close to .5 in Canada and the US, on average the scales in every country fall below .5 (see Table 1). This reproduces previous results in Canada (Banting et al., 2020; Harell, Banting, Kymlicka, & Wallace, 2022) using an alternative, comparative wording with slightly different items.^18^ The comparison across countries is noteworthy. Membership perceptions are least negative toward immigrants in the US, followed by Canada. Scores in Europe are consistently more negative, though to a lesser extent in Great Britain.

**Table 1.** Attitudes Toward Immigrants and the Nation.

	US	CA	GB	FR	IT	DK	SE
Membership	0.49	0.45	0.40	0.32	0.32	0.34	0.33
Ethnic nationalism	0.44	0.40	0.49	0.45	0.37	0.47	0.37
Civic nationalism	0.88	0.88	0.86	0.89	0.91	0.90	0.91
National ID	0.83	0.82	0.75	0.84	0.79	0.80	0.80
Immigrants’ control	0.52	0.50	0.49	0.55	0.58	0.55	0.58
Immigrants’ need	0.53	0.51	0.55	0.53	0.59	0.54	0.55
Affect toward immigrants	0.55	0.54	0.46	0.41	0.53	0.37	0.48
Anti-immigrant scale	0.43	0.42	0.45	0.55	0.52	0.52	0.55

*Note.* Scores are standardized from 0-1. Excludes foreign-born. Weighted.

To give a sense of the extent of these negative perceptions, about 49% of the native-born sample in Canada and about 43% in the US accord immigrants mean scores below the midpoint. In Great Britain, almost 60% of the native-born sample accord immigrants scores below this threshold, rising to three quarters – a clear super majority (between 72-76%) - in the remaining European countries.^19^ In all cases, the distribution is skewed toward more negative assessments.

Table 1 provides the mean scores for membership, as well as the other attitudinal factors included in this study. As we noted earlier, some may suggest that membership perceptions are really just a manifestation of more familiar and well-studied attitudes, such as civic versus ethnic nationalism, outgroup antipathy, or beliefs about hard-work/laziness (called “control” in the deservingness literature). As Table 1 makes clear, membership perceptions do not neatly track any of these other attitudes. Countries with similar (high) levels of civic nationalism, for example, vary greatly in perceived membership of immigrants. Similarly, countries with similar levels of belief about immigrant responsibility for poverty have significantly different perceived membership scores. Moreover, Table 1 also reveals that the variation in membership perceptions between countries is larger than the variation in most other attitudinal variables. This suggests that membership perceptions form a distinct basis for evaluating the claims of immigrants. While we expect various assessments of immigrant deservingness to be intercorrelated, Table 1 makes evident that at the aggregate level, the ranking of countries shifts depending on what attitudinal dimension we focus on, and further, that membership perceptions vary in unique ways across these countries.^20^

In short, while there is some cross-national variation in the size of these membership penalties, in all seven countries immigrants are seen as having lower membership commitment. A sizeable portion of the general public, and indeed important majorities in most countries, view immigrants as less committed to and concerned about the political community in which they now live.

We turn next to exploring the extent to which these membership perceptions influence attitudes toward redistribution. Our analytic strategy is to assess the nature and size of the effect of membership perceptions in each country, in part compared to more classic explanations. Recall that our main interest is in how membership perceptions affect support for inclusive redistribution: that is, support for including immigrants in general social programs (as against various forms of “welfare chauvinism”). However, we begin by exploring whether membership perceptions affect attitudes toward redistribution more generally. Does viewing immigrants as committed to the larger society make respondents more supportive of the welfare state in general?

**Figure 2.**
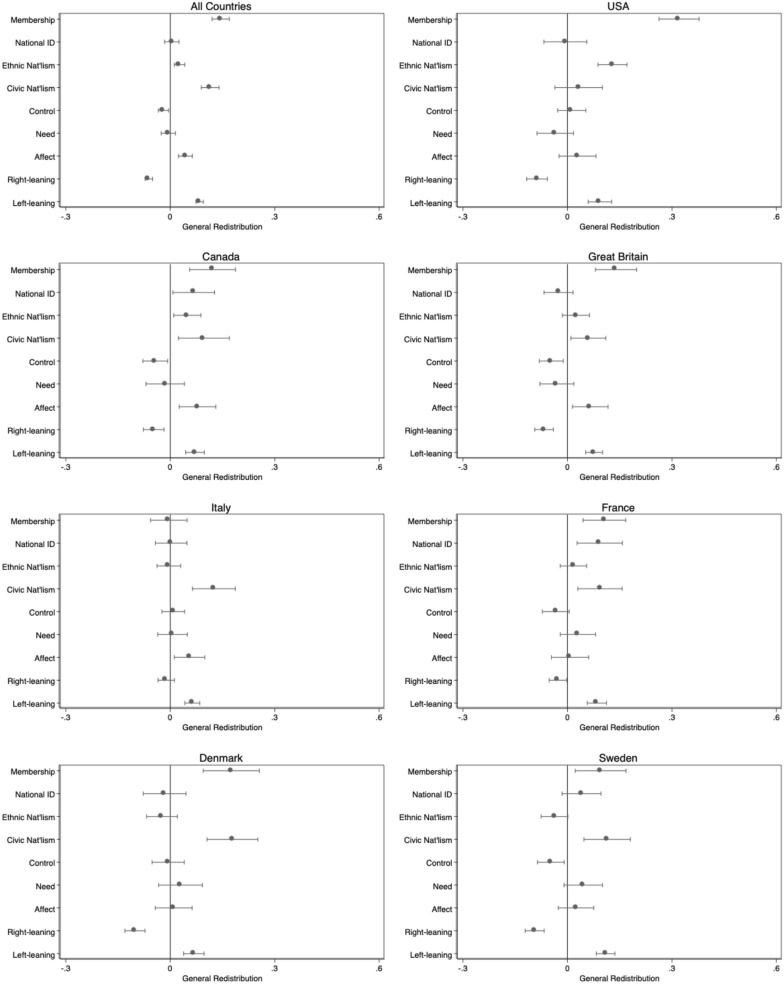
Predicted levels of general redistribution by country and membership perceptions. Note: For simplicity, the figure excludes some control variables. Based on full models presented in Appendix Table B1, including a control for foreign-born. 95% confidence intervals. Weighted.

Previous studies on this have reached mixed results. Harell, Banting, Kymlicka, and Wallace (2022) found no clear relationship between membership attitudes and support for general redistribution in Canada.^21^ Sandelind and Hjerm (2022), however, found a positive effect of perceived immigrant commitment on support for general redistribution in Sweden. Figure 2 presents the average marginal effect of a one unit increase in membership perceptions alongside our competing attitudinal variables. Full models, including sociodemographic controls, are reported in the Appendix. What we see is that membership perceptions of immigrants are significant in the pooled model, but their impact varies across countries. The impact is greatest in the US, with significant but smaller effects elsewhere, and no relationship evident in Italy.

We note that our single item measure of civic nationalism tends to be as or more powerful, with the exception of the United States, where *ethnic* nationalism has a noteworthy effect. We find little evidence that the strength of national identity matters, though it is significant in France and Canada. Results are inconsistent across countries for other variables, though perceptions of immigrants as lazy (control) tend toward negative and more positive affect in a few cases increases support. Finally, ideology functions as expected with left-leaning respondents more supportive of redistribution and right-leaning respondents less, compared to those in the centre.

**Figure 3.**
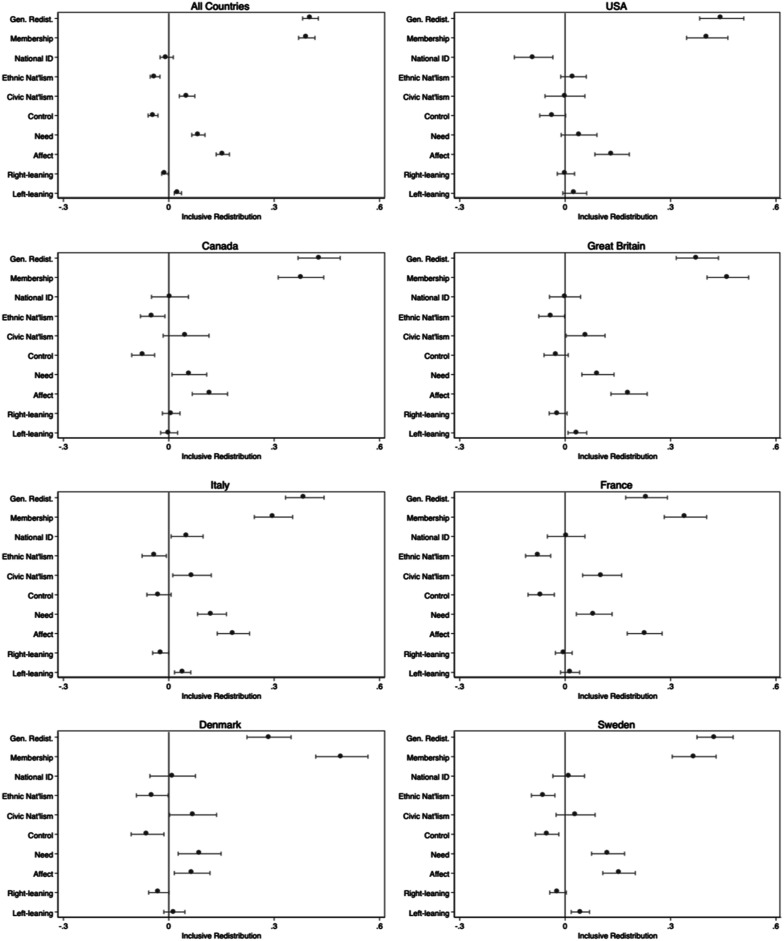
Average marginal effects of key attitudinal predictors of inclusive redistribution. Note: For simplicity, the figure excludes some control variables. Based on full models provided in Appendix Table B2, including a control for foreign-born. 95% confidence intervals shown on estimates. Weighted.

Our main focus, however, is the role of membership perceptions in shaping inclusive redistribution – i.e., support for the inclusion of immigrants within core redistributive programs. We strongly suspect that membership will be a powerful predictor of support for inclusive redistribution, which we turn to in Figure 3 which presents the average marginal effects on *inclusive* redistribution. We add here a control for people’s general support for redistribution because we want to isolate general support for the welfare state from more inclusive – or less chauvinistic – attitudes. As is evident in Figure 3, membership perceptions are consistently one of the strongest additional predictors, sometimes even stronger than general attitudes toward redistribution (Full models with sociodemographic controls in Appendix 2A). This effect is observed in both the pooled model and in each country in the sample.

This is not to say that other attitudes do not also matter. Not surprisingly, those with less negative affect toward immigrants (as measured by a feeling thermometer) tend to support more inclusive redistribution. We find the same effect when we capture xenophobic attitudes using an alternative anti-immigrant scale (see Appendix, Table A4). In every country but the US, ethnic nationalism is negatively related to inclusive redistribution. In the US, in contrast, a strong national identity decreases support for inclusive redistribution but has no discernible effect in other countries. Control (the belief that immigrants are poor because they are lazy) tends to have an expected negative effect, though it is not always significant. A recognition of a greater need (immigrants being less well-off than others) tends to push support up, except in the US. Yet, the belief that immigrants care about others and are willing to sacrifice as much or more than others in their new country - in other words that they are perceived to be committed members of the political community – overwhelmingly creates a context in which native-born citizens support their inclusion within the welfare state.

**Figure 4.**
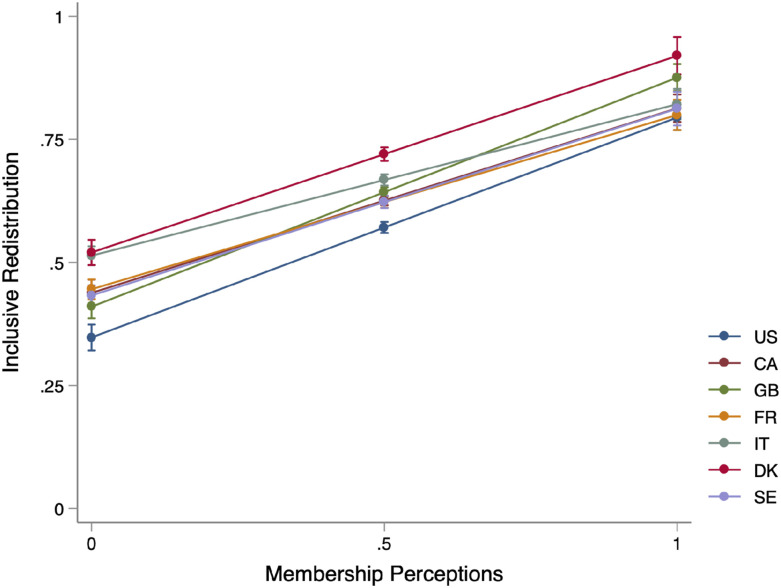
Predicted levels of inclusive redistribution by country and membership perceptions. Note: Figure presents the predicted levels of inclusive redistribution across the membership scale. Full models, which included controls and an interaction between membership and country, are available in Appendix B4. 95% confidence intervals. Note models control for foreign-born. Weighted.

We can illustrate the size of this effect in each country in a pooled model where we interact the membership scale with country. Figure 4 provides the predicted level of inclusive redistribution in each country as perceptions of membership improve (See Appendix Table B4 for full model). The direction of the effect is consistently positive. While the slope of the relationship varies somewhat across countries, the overall pattern is a consistently positive relationship. This suggests that the effect of membership perceptions on support for inclusive redistribution is largely generalizable across these countries.

Recall that the average score on the membership scale varies between .32 and .49 in the seven countries in our sample, and in every case is skewed toward negative evaluations with between 4% and 14% of the country samples having the lowest possible score of 0. Recall that in Figure 3, the substantive size of the effect of membership perceptions was comparatively large compared to some of the usual suspects. If we move from negative attitudes (0) to the midpoint of equal commitment (which is approximately two standard deviations), Figure 4 shows an increase between, on the low end, .15 (IT) and .18 (FR) to a high of .22 (US) and .23 (GB) on the 0 to 1 inclusive redistribution scale. Substantively, that means about a one category shift upwards on the original five-point scale on which inclusive redistribution was measured, and in most cases shifts support for inclusive redistribution from negative or neutral (.5) score to a positive one (>.5). Substantively, then, this reinforces the conclusion that membership perceptions are powerful in predicting inclusive redistribution, and this is the case across the seven countries in our sample.

## Discussion and Conclusion

Perceptions of immigrant’s membership in the political community are powerful predictors of support for redistribution, and particularly a form of redistribution that includes immigrants within the core benefits of the welfare state. While much of past research has focused on economic and cultural factors that define immigrants as undeserving of such benefits, we develop an alternative theoretical approach that focuses on conceptually – and in turn measuring – the extent to which immigrants are viewed as full, committed members of the national “we”. While seeing immigrants as an economic threat to the nation, or as culturally distinct outgroups, certainly hinders more inclusive forms of redistribution, we argue here that support of a generous welfare state that includes immigrants within its programs requires a belief that immigrants are part of the circle of solidarity, which requires reciprocity that extends beyond just working and paying taxes, something that previous work has highlighted. It also requires a more affective component based on mutual caring and a willingness to sacrifice for the collectivity, and indeed our work shows that ‘contributing’ can take many forms and is not simply a tit for tat economic exchange. This form of reciprocity between native-born citizens and immigrants is, in our opinion, also not simply a result of the elimination of prejudicial attitudes. A belief that immigrants are equally committed to the political community reflects an acceptance of them as full members of a given society, and a belief they are willing to take up the obligations of that inclusion.

Importantly, we do not suggest that these perceptions are necessarily accurate; nor are we suggesting that such perceptions are appropriate normative grounds for determining whether immigrants deserve inclusion. Undoubtedly, various forms of bias may enter into these perceptions, and reducing prejudicial attitudes is likely a necessary condition for promoting more positive assessments of immigrants’ membership. However, our evidence suggests that reducing prejudice is insufficient on its own. Neither are simple endorsements of democratic values and institutions, nor the strength of one’s own identity with the country. The story of when people are willing to share the wealth of a society with newcomers is incomplete if we focus solely on eliminating prejudicial attitudes, the level of need within these communities, or citizens’ own attachment to the country. Native-born citizens are more likely to accept obligations towards newcomers when they believe that newcomers have made a commitment to the larger society in which they now reside.

This is a good news, bad news story. The bad news is that immigrants (and other minorities) may be asked to prove or perform their “commitment” to gain access to rights that majorities take for granted, and it is likely that even when they do perform such a commitment, they are subject to double-standards (e.g., having to meet a higher bar of commitment or sacrifice). The good news, however, is that these membership perceptions are not cast in stone, and some societies have significantly lower membership penalties (without thinning or weakening general redistribution). And these lower penalties may be the result of public policies that signal public confidence in minorities’ commitment, and thereby reduce the burdens on immigrants to prove it. A crucial area of future research, therefore, is what factors reduce membership penalties, and, in particular, how public institutions and policies can create a more level playing field for majorities and minorities to claim membership.

Membership, in this sense, provides an alternative conception of the political community that is distinct from debates around ethnic and civic forms of nationalism. When we view the political community as a set of overlapping obligations to those within its circle, the question becomes who we include within that circle. Newcomers, and other minoritized groups, are often seen as being outside of the circle, despite often attaining formal status as legal residents or citizens. Our study makes clear, across a variety of countries in North America and Europe, that when native-born citizens see immigrants as committed members who care about others in their host society and are willing to contribute and make sacrifices for it, they are much more likely to support a more robust inclusion of immigrants in redistribution.

Our study is not without its limits. The category of “immigrant” is very general and likely to be understood somewhat differently in each country under study here. Who people think of when they hear the word ‘immigrant’ likely reflects differences in their country’s immigration history, but also in individuals’ social contexts. Indeed, future studies can and should explore how people view *specific* immigrant groups, and we would expect variation across salient groups. Yet immigration as a policy domain is often discussed more generally in public debates. While we are not able to speak to the specific types of immigrants that people have in mind in each country, we know 1) that citizens vary in their assessments of immigrants’ membership, 2) these assessments on average are negative across a variety of countries, and 3) negative membership assessments are correlated with less support for redistribution, especially inclusive redistribution.

Our study is also limited in so far as we can document the relationship between these attitudes but are limited in teasing out the causal direction with correlational survey data. Yet recent work suggests that these attitudes are malleable and that manipulating signals of membership commitment can improve perceptions of commitment and increase support for redistribution (Harell et al. nd). More needs to be done to explain why membership levels vary across countries and the individual factors that lead native-born citizens to view immigrants as more or less committed. As we noted, we suspect that some institutional factors such as the robustness of integration regimes as well as the nature of the redistribution programs in a country may provide some leverage. There is no doubt that individuals’ experiences with immigrants may also play a role. What we hope to have made clear in this paper is that membership penalties appear to be an important attitudinal barrier to public support for inclusive welfare regimes.

## Supplemental Material

Supplemental Material - Inclusive Redistribution and Perceptions of Membership: A Cross-National Comparison Supplemental Material for Inclusive Redistribution and Perceptions of Membership: A Cross-National Comparison by Allison Harell, Keith Banting, and Will Kymlicka in Comparative Political Studies

## Data Availability

Harell, A, Banting, K, & Kymlicka, W. (2025) Replication Data for: Inclusive Redistribution and Perceptions of Membership: A Cross-National Comparison, https://doi.org/10.7910/DVN/W6JE2K, Harvard Dataverse, V1.

## Notes

1. Though note that Sobolewska and colleagues (2017) do show that language acquisition and voting are positively related to evaluating immigrants as more integrated.

2. Such a view reflects T.H. Marshall's famous argument that the welfare state rests on “a direct sense of community membership based on loyalty to a civilization that is a common possession” (1950, p.96). For Marshall – and arguably for the social democratic tradition more generally – the welfare state is rooted in an ethic of social membership. It presupposes that there is such a thing as a shared “society”; that this society is seen by its members as their “common possession” towards which they have distinctive rights and responsibilities; and that the welfare state is intended to enable members to participate in, enjoy and help shape their shared society as a common possession. For an application of Marshall’s ideas to debates on inclusive solidarity, see Kymlicka 2022.

3. In places, Miller acknowledges that inclusive national solidarity requires not only that majorities endorse a civic conception of the nation but also that they come to perceive minorities as sharing that commitment (Miller, 2017, p. 73; Miller, 2006, p. 332). However, when it comes to testing his theory, he only focuses on the first part (i.e, the strength of respondents’ own sense of national identity). We argue that the real action is around the second part (i.e., whether majorities perceive minorities as sharing a sense of loyalty and commitment).

4. Perceptions of membership also appear to predict political solidarity and support for civil rights (Scott, 2023; see also Verkuyten et al., 2022).

5. We also did a more preliminary and limited test of the relationship between membership perceptions and support for inclusive redistribution in a survey conducted in nine countries in 2020. The results confirmed the core relationship, though with a smaller battery of membership perception questions and fewer controls (Harell, Kymlicka & Banting, 2022).

6. We note that the surveys included a second group in each country (Black nationals in Canada and the US and Muslims in the European countries). These questions are not the focus of this paper.

7. Each survey was in the field for 7–17 days. Respondents completed a captcha before being able to complete the survey. Post data collection, poor quality respondents were removed from final datasets (ranging from 3.1% 7.3% of the respondents who completed the full survey). Poor quality data included those who completed the survey in less than a third of the median survey time, or those who failed at least two quality control checks in the survey (excessive DK responses, straight-lining balanced scales, and/or failed an attention check). Further details about survey fielding are available in Appendix Table A1.

8. Post weights were created using an iterative ‘raking’ process using the *ipfraking* command in Stata based on demographic quota variables. A maximum of 200 iterations was permitted.

9. Though note that Southern states in Europe like Italy are sometimes classified separately from the classic continental models such as France (Rhodes, 1996).

10. Cronbach’s alpha = .74 in pooled sample and varies between .66 and .78 in each country. Pairwise deletion was used in the creation of the scale.

11. The index has a Cronbach’s alpha = .80 in pooled sample with similar scores when calculated separately by country (varying between .79 and .82). Pairwise deletion was used in the creation of the scale.

12. Previous work has used a similar measure. See Banting et al., 2020; Harell, Banting, Kymlicka, & Wallace, 2022; Scott 2023.

13. Note that following the questions focused on immigrants, respondents also answered questions about one other group in each country (Black nationals in Canada and the US; Muslims in the country in Europe).

14. The survey also included a five item anti-immigrant scale. Given that anti-immigrant scales tend to include items that capture people’s perceptions of the consequences of immigration, rather than attitudes about immigrants themselves, the feeling thermometer is a more direct measure of affect. That being said, we provide evidence in the Appendix that the results are robust to using alternative measures of prejudice (See Appendix Table B3).

15. The three categories were created based on an eleven-point left-right self-placement question from 0-10. Scores of 0–3 were coded as left-leaning, 4-6 as centrist, and 7-10 were coded as right-leaning. Note that don’t know and missing responses were coded in the centrist category.

16. Replication materials and code can be found at Harell et al. (2025).

17. Average responses for each individual item in the scale by country are available in the Appendix Figure A1.

18. Note that in the previous study in Canada, respondents assessed immigrants and English-speaking Canadians separately, and the difference in scores was used to assess the penalty. In the current study, we use a more general reference category (“other [nationals]”) and the question is explicitly comparative. This change in format makes the questions less onerous in terms of survey space, while also ensuring that the reference category are others in society.

19. Note that these percentages are based on weighted distributions, with foreign-born respondents excluded.

20. While the focus of this paper is not to explain the cross-national differences, but rather to document the extent to which membership penalties exist across countries and their impact on redistribution attitudes, we have included in Appendix C some basic information about the immigrant and institutional context in each country. As noted earlier, one possible factor that might affect perceived membership is a country’s naturalization and multiculturalism policies, insofar as liberal policies signal confidence about immigrants’ potential membership. The data in Appendix C provide some support for this. Based on the Migrant Integration Policy Index (MIPEX), we see that Italy, France, Denmark and the UK are relatively low, with Canada and the US with higher scores. Similarly, if we draw on the Multiculturalism Policy Index (MPI), Canada and the UK score highly whereas Denmark, France and Italy have low scores. Yet in both cases, Sweden defies the pattern: it scores highly in terms of MIPEX and MPI, yet has low levels of perceived membership commitment. The link between citizenship/multiculturalism policies and membership perceptions is an important topic for future research.

21. Note that this study used a different version of the membership scale where immigrant scores were subtracted from membership scores for English-speaking Canadians. The current measure asks for direct comparisons of immigrants’ membership compared to “other [nationals]”.
