# Supplementary material for: Inclusive Redistribution and Perceptions of Membership: A Cross-National Comparison
Source: Comp Polit Stud. 2025 May 27;59(4):803–30. doi: 10.1177/00104140251342924 (PMC12863465; doi:10.1177/00104140251342924)
Supplement: Supplemental Material - Inclusive Redistribution and Perceptions of Membership: A Cross-National Comparison [file sj-pdf-1-cps-10.1177_00104140251342924.pdf]

Supplemental Material

Allison Harell, Keith Banting & Will Kymlicka (2025) Inclusive Redistribution and Perceptions of Membership: A Cross-National Comparison, *Comparative Political Studies*

Appendix A: Overview of Data Collection Dates, Sample Characteristics and Coding

Provider for all panel data was Cint, with representative quotas for age, gender, education and region. A language quota was added to Canada. The survey was fielded in the primary language of each country (English in the US, and Great Britain, French in France, Danish in Denmark, Swedish in Sweden and in both English and French in Canada, where respondents were allowed to answer in the language of their choice). In each case, the translation was done by a native speaker of the language, and reviewed and approved by a team member from each case country for equivalence with the master English version.

Target population was permanent residents and citizens 18 years or older, currently living in the country. To be eligible, respondents also needed to meet certain quality control checks. Low quality respondents include those who completed the survey in less than a third of the median survey time, or those who failed at least two quality control checks in the survey (excessive DK responses, straight-lining balanced scales, and/or failed an attention check).

Weights were created post data collection using an iterative 'raking' process using the *ipfraking* command in Stata based on demographic quota variables. A maximum of 200 iterations was permitted.

Table A1: Data Collection Dates and Sample Characteristics

Country	*Completed Survey (N entered)*	*Final Clean N*	*Removed for Low Quality*	*Field Dates*	*Median Survey Length (min)*
Canada	2142 (3184)	2046	96	Nov. 15-Nov. 22, 2021	18.6
USA	2175 (4802)	2020	155	Dec. 8-Dec. 21, 2021	17.7
Great Britain	2077 (4148)	2006	72	Jan. 28-Feb. 7, 2022	16.7
Italy	2100 (8,942)	2033	67	Feb. 25-3 Mar., 2022	19.9
France	2000 (3348)	1898	102	Jun. 22-Jul. 5, 2022	21.5
Sweden	2006 (3033)	1894	112	Jun. 23-Jul. 7, 2022	18.6
Denmark	2010 (3228)	1863	147	Jun. 23-Jul. 10, 2022	19.1

We report in Table A1 we provide further information on the final sample in each country, including the number of respondents who completed the survey. Not that the number of respondents who entered a survey is always larger than the number who completed the survey. This includes respondents who simply landed on the consent page and did not continue, but also, and primarily, respondents who were invited but responded in the first question bloc as being from a quota that was already completed. They were then prevented from completing the survey. The number was particularly high in Italy, where the panel provider had more difficulty filling the regional quotas in our study.

Data Availability and Ethics

Data and code to reproduce this article can be found here:

Harell, Allison; Keith Banting; Will Kymlicka, 2025, "Replication Data for: Inclusive Redistribution and Perceptions of Membership: A Cross-National Comparison", https://doi.org/10.7910/DVN/W6JE2K, Harvard Dataverse, V1

Data collection was conducted with ethics approval through the Université du Québec à Montréal (#2022-3386, 4430).

Question Wording and Variable Creation

Coding of variables for the analysis are described below.

General Redistribution: Based on the following three questions, standardized on a 0-1 additive scale with pairwise deletion. Original responses were on a five point agree-disagree scale.

1) The government should provide social assistance for those in need.
2) The government should see to it that everyone has a decent standard of living.
3) Government should redistribute income from the better-off to those who are less well off.

Inclusive Redistribution: Based on the following three questions, standardized on a 0-1 additive scale with pairwise deletion. Original responses were on a five point agree-disagree scale.

Thinking about **immigrants in Canada**, do you agree or disagree that it should be the government's responsibility to…

1) ensure immigrants have access to social assistance when they need it.
2) provide a decent standard of living for immigrants in (country).
3) reduce income differences between immigrants and other (nationals).

Membership Commitment: Based on the following questions, standardized on a 0-1 additive scale with pairwise deletion. Original responses were on a five point much less to much more scale.

Now we would like to ask you some questions about various groups in Canada. First, we want you to think about **immigrants in general.**

1) Compared to other [nationals], how much do you think immigrants identify with the country?
2) Compared to other [nationals], how much do you think immigrants care about the concerns and needs of other [nationals]?
3) Compared to other [nationals], how willing do you think immigrants are to make sacrifices for others in our society?
4) Compared to other [nationals], do you think immigrants are contributing their fair share by working and paying taxes, or more or less than their fair share?

5) Compared to other [nationals], how proud do you think immigrants are to be [national]?
6) If [country] was involved in a war, how willing do you think immigrants would be to volunteer to fight for the country, compared to other [nationals]?

Ethnic Nationalism: Some people say that the following things are important for **being truly [national]**. Others say they are not important. How important do you think each of the following is? To have [national] ancestry (Four point scale from Not at all important to very important)

Civic Nationalism: Some people say that the following things are important for **being truly [national]**. Others say they are not important. How important do you think each of the following is? To respect [country]'s political institutions and laws (Four point scale from Not at all important to very important)

National Identity: Additive scale of two items:

1) How strongly do you identify as a [national]? (four point scale from very strongly to not strong at all)
2) How proud are you of being [national]? (four point scale from very proud to not proud at all)

Immigrants' Control: **Immigrants** in this country sometimes face economic hardship. Here are four possible reasons why. Please tell us how important each reason is in explaining economic hardship among **immigrant communities** in this country. Because of laziness and lack of willpower (Four point scale from Not at all important to very important)

Immigrants' Need : Are immigrants better or worse off than other Canadians? (Five point scale from much less to much better).

Affect toward Immigrants : Now we would like to know what your general feelings are about different groups in Canada. Please rate how close or distant you feel to the ideas and interests held by the group, where 0 means very distant and 100 means very close. "Immigrants" (Scale from 0 very distant to 100 very close).

Ideology : Item recoded into three categories (0-3 left; 4-6 centre, 7/10 right) with don't know responses recoded as centre/ambivalent.

In politics, people sometimes talk of left and right. Where would you place yourself on this scale?

Gender: Are you: a man, a woman, non-binary, not-listed (please indicate). Recoded where man=1, woman or non-binary=0.

Income: What was your total household income, before taxes, for the year 2020? Be sure to include income from all sources. Scales differed in each from based on common income brackets in local currency. Income was recoded to vary from 0-1 on the full scale asked in each country.

Education: What is the highest level of education that you have completed? Response categories were specific to each country's educational system, and recoded to be 0 (less than university) 1 (BA or more)

Foreign-born: Were you born in [country]? Yes (0), No (1)

Age: First, how old are you? Responses range from 18 to 90 plus.

Religion: What is your religion, if you have one? Religion affiliation list varied by country and recoded to be 0=not religious; 1 "Christian denomination"; 2 "non-Christian denomination.

Anti-Immigrant Scale : Based on the following questions, standardized on a 0-1 additive scale with pairwise deletion. Original responses were on a five point agree-disagree scale.

1) Immigration is good for [country]'s economy. (reverse coded)
2) Too many recent immigrants just don't want to fit into [national] society.
3) Immigrants take jobs away from other [nationals].
4) [Country]'s cultural life is enriched by immigrants to this country. (reverse coded).
5) Immigrants increase crime rates in [country].

Table A2: Sample Characteristics

	Unweighted Sample	OECD 2022 Population	Difference (Sample-Pop)
**Gender (% Men)** [a]			
Canada	45.2	49.7	-4.5
Denmark	50.1	49.7	0.4
France	49.7	48.4	1.3
Italy	47.7	48.7	-1
Sweden	49.2	50.3	-1.1
United Kingdom	46.8	49.4	-2.6
United States	47.8	49.6	-1.8
**Educational Attainment (BA or greater for ages 24-65)** [b]			
Canada	35.3	34.0	1.3
Denmark	38.6	35.7	2.9
France	35.6	32.3	3.3
Italy	24.4	18.0	6.4
Sweden	33.5	29.6	3.9
United Kingdom	42.6	38.4	4.2
United States	39.2	37.3	1.9
**Age** [b]			
Canada			
18-34	25.9	25.8	0.1
35-54	40.5	33.1	7.4
55+	33.6	41.1	-7.5
Denmark			
18-34	28.3	27.5	0.8
35-54	28.8	31.1	-2.3
55+	42.9	41.4	1.5
France			
18-34	31.6	25	6.6
35-54	33.14	32.3	0.8
55+	35.3	42.8	-7.5
Italy			
18-34	22.0	20.9	1.1
35-54	39.2	34.5	4.7
55+	39.8	44.6	-4.8
Sweden			
18-34	29.3	27.6	1.7
35-54	33.9	32.1	1.8

55+	36.9	40.3	-3.4
United Kingdom			
18-34	28.6	29.0	-0.4
35-54	35.1	35.2	-0.1
55+	36.3	35.8	0.5
United States			
18-34	26.4	29.6	-3.2
35-54	32.7	32.6	0.1
55+	40.8	37.7	3.1
**Foreign Born** [b]			
Canada	20.3	21.0	-2.7
Denmark	6.3	10.5	-4.2
France	5.7	12.8	-7.1
Italy	2.9	10.4	-7.5
Sweden	13.4	19.5	-6.1
United Kingdom	9.7	13.7	-7.1
United States	5.9	13.6	-7.7

[a] Gender was extracted from the World Bank Data Bank on Gender Statistics. 2020 data reported.
[b] Extracted from national statistics agencies and OECD.stat. Notre for education attainment, estimate is for 24-65 age group only. The sample characteristics reported in the table are limited to that age group for the university education variable. 2019-2022 based on availability.

Figure A1: Average Membership Scores by Country by Item

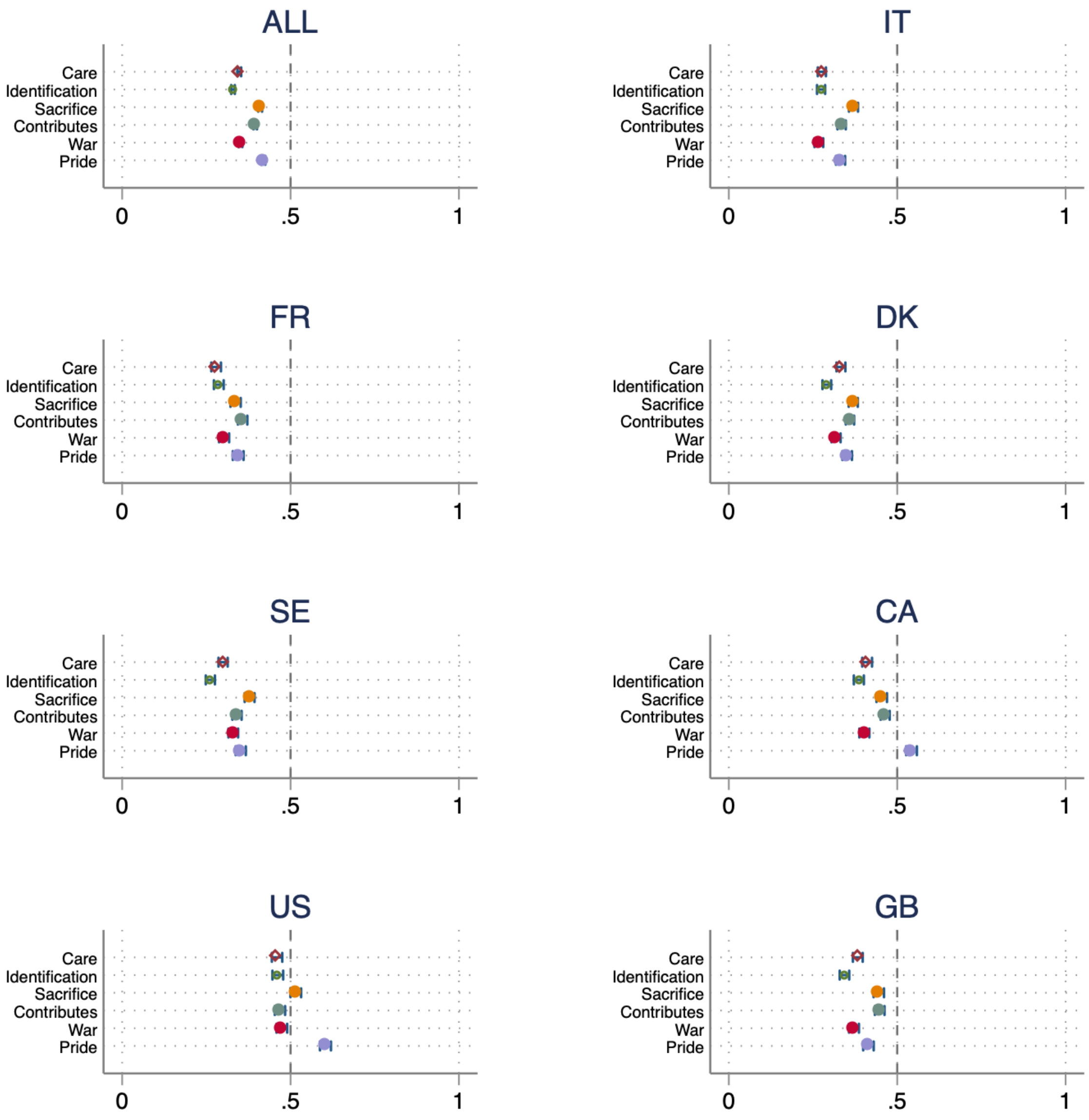

95% Confidence Interval, Limited to non-foreign born, weighted

Appendix B: Full Models for Figures in Article and Robustness Checks

Table B1: Separate models for each country explaining general redistribution (Figure 2)

	All	US	CA	GB	IT	FR	DK	SK
Membership	0.145***	0.320***	0.121***	0.139***	-0.004	0.106***	0.175***	0.095*
	(0.013)	(0.029)	(0.034)	(0.030)	(0.027)	(0.031)	(0.041)	(0.037)
National ID	0.004	-0.006	0.067*	-0.026	0.003	0.093**	-0.016	0.040
	(0.011)	(0.031)	(0.030)	(0.021)	(0.023)	(0.033)	(0.031)	(0.028)
Ethnic nat'l	0.027***	0.129***	0.049*	0.024	-0.004	0.017	-0.024	-0.037
	(0.008)	(0.021)	(0.020)	(0.020)	(0.017)	(0.019)	(0.023)	(0.020)
Civic nat'l	0.115***	0.032	0.097**	0.060*	0.125***	0.093**	0.178***	0.114***
	(0.013)	(0.035)	(0.037)	(0.026)	(0.031)	(0.032)	(0.037)	(0.034)
Immigrants' Control (lack)	-0.020**	0.012	-0.043*	-0.046**	0.009	-0.034	-0.006	-0.048*
	(0.008)	(0.021)	(0.018)	(0.018)	(0.017)	(0.020)	(0.023)	(0.020)
Immigrants' Level of Need	-0.006	-0.035	-0.015	-0.030	0.007	0.030	0.029	0.045
	(0.010)	(0.027)	(0.028)	(0.025)	(0.022)	(0.026)	(0.032)	(0.028)
Thermometer (Affect)	0.043***	0.029	0.078**	0.065*	0.055*	0.007	0.010	0.025
	(0.010)	(0.027)	(0.027)	(0.026)	(0.022)	(0.027)	(0.027)	(0.026)
Right-leaning	-0.062***	-0.087***	-0.048**	-0.067***	-0.012	-0.027*	-0.101***	-0.094***
	(0.005)	(0.015)	(0.015)	(0.014)	(0.012)	(0.013)	(0.015)	(0.014)
Left-leaning	0.084***	0.093***	0.071***	0.077***	0.063***	0.085***	0.068***	0.110***
	(0.005)	(0.017)	(0.014)	(0.012)	(0.011)	(0.014)	(0.015)	(0.014)
Man	-0.012**	-0.019	0.005	0.010	-0.014	0.002	-0.024	-0.039**
	(0.004)	(0.013)	(0.011)	(0.010)	(0.010)	(0.011)	(0.012)	(0.012)
Christian	-0.009	-0.011	-0.015	-0.003	0.014	-0.023*	-0.012	-0.005
	(0.005)	(0.016)	(0.012)	(0.011)	(0.012)	(0.012)	(0.014)	(0.012)
Other religions	-0.011	0.020	-0.021	-0.001	0.070	0.001	0.015	-0.073*
	(0.009)	(0.021)	(0.019)	(0.019)	(0.047)	(0.025)	(0.025)	(0.029)
Foreign born	-0.016*	-0.026	0.002	-0.028	0.018	0.002	-0.035	-0.039*
	(0.008)	(0.027)	(0.014)	(0.018)	(0.030)	(0.025)	(0.029)	(0.020)
University Degree	-0.011*	-0.028	0.009	-0.003	-0.008	-0.007	0.002	-0.002
	(0.005)	(0.014)	(0.012)	(0.011)	(0.011)	(0.013)	(0.013)	(0.013)
Income	-0.062***	-0.065*	-0.155***	-0.101***	-0.034	-0.118***	-0.024	-0.075**
	(0.010)	(0.029)	(0.027)	(0.024)	(0.020)	(0.035)	(0.022)	(0.024)
Age	0.000	-0.002***	-0.001***	-0.000	0.000	0.001**	0.001	0.002***
	(0.000)	(0.000)	(0.000)	(0.000)	(0.000)	(0.000)	(0.000)	(0.000)
DK	-0.047***							
	(0.009)							
FR	0.010							
	(0.008)							
GB	0.040***							
	(0.008)							
IT	0.075***							
	(0.008)							
SK	-0.028**							

	(0.009)							
US	-0.051***							
	(0.009)							
Constant	0.602***	0.572***	0.633***	0.750***	0.651***	0.553***	0.499***	0.564***
	(0.018)	(0.044)	(0.045)	(0.038)	(0.039)	(0.045)	(0.054)	(0.047)
N	9684	1432	1430	1369	1450	1387	1183	1433

Robust standard errors in parentheses
* $p < 0.05$, ** $p < 0.01$, *** $p < 0.001$

Table B2: Separate models for each country explaining inclusive redistribution (Figure 3)

	All	US	CA	GB	IT	FR	DK	SK
Gen. Redist.	0.403^{***}	0.446^{***}	0.428^{***}	0.376^{***}	0.387^{***}	0.232^{***}	0.285^{***}	0.427^{***}
	(0.011)	(0.032)	(0.031)	(0.031)	(0.028)	(0.030)	(0.032)	(0.026)
Membership	0.393^{***}	0.405^{***}	0.376^{***}	0.463^{***}	0.298^{***}	0.343^{***}	0.493^{***}	0.367^{***}
	(0.012)	(0.030)	(0.033)	(0.030)	(0.028)	(0.031)	(0.038)	(0.032)
National ID	-0.006	-0.090^{**}	0.003	-0.000	0.052^{*}	0.003	0.011	0.010
	(0.010)	(0.028)	(0.027)	(0.023)	(0.023)	(0.027)	(0.033)	(0.023)
Ethnic nat'l	-0.039^{***}	0.024	-0.046^{**}	-0.038^{*}	-0.041^{*}	-0.077^{***}	-0.047^{*}	-0.062^{***}
	(0.007)	(0.019)	(0.018)	(0.019)	(0.018)	(0.018)	(0.023)	(0.017)
Civic nat'l	0.052^{***}	-0.000	0.049	0.058^{*}	0.066^{*}	0.105^{***}	0.069^{*}	0.030
	(0.011)	(0.029)	(0.033)	(0.028)	(0.028)	(0.028)	(0.034)	(0.028)
Immigrants' Control (lack)	-0.045^{***}	-0.035	-0.073^{***}	-0.025	-0.028	-0.068^{***}	-0.061^{*}	-0.051^{**}
	(0.007)	(0.019)	(0.017)	(0.018)	(0.018)	(0.019)	(0.024)	(0.017)
Immigrants' Level of Need	0.084^{***}	0.040	0.058^{*}	0.093^{***}	0.123^{***}	0.083^{**}	0.088^{**}	0.122^{***}
	(0.010)	(0.026)	(0.025)	(0.023)	(0.021)	(0.026)	(0.031)	(0.024)
Thermometer (Affect)	0.154^{***}	0.134^{***}	0.117^{***}	0.182^{***}	0.184^{***}	0.226^{***}	0.066^{*}	0.154^{***}
	(0.010)	(0.025)	(0.026)	(0.026)	(0.023)	(0.025)	(0.026)	(0.024)
Right-leaning	-0.011^{*}	0.002	0.007	-0.020	-0.023^{*}	-0.004	-0.028	-0.020
	(0.005)	(0.013)	(0.013)	(0.013)	(0.012)	(0.012)	(0.015)	(0.012)
Left-leaning	0.026^{***}	0.027	0.001	0.035^{*}	0.039^{***}	0.014	0.016	0.043^{**}
	(0.005)	(0.017)	(0.012)	(0.014)	(0.012)	(0.014)	(0.015)	(0.013)
Man	-0.014^{***}	0.011	-0.002	-0.015	-0.020^{*}	-0.004	-0.040^{**}	-0.027^{**}
	(0.004)	(0.011)	(0.010)	(0.010)	(0.009)	(0.010)	(0.013)	(0.010)
Christian	0.000	0.024	-0.017	0.001	-0.019	-0.000	0.001	0.002
	(0.004)	(0.014)	(0.011)	(0.011)	(0.011)	(0.011)	(0.014)	(0.010)
Other religions	0.004	0.031	-0.019	0.003	-0.007	0.008	0.014	-0.007
	(0.008)	(0.018)	(0.016)	(0.019)	(0.055)	(0.021)	(0.023)	(0.019)
Foreign born	-0.000	0.007	0.007	0.014	-0.024	-0.001	-0.010	-0.010
	(0.007)	(0.025)	(0.013)	(0.018)	(0.032)	(0.021)	(0.028)	(0.015)
University Degree	0.011^{**}	0.016	0.015	0.006	-0.004	0.025^{*}	0.034^{**}	-0.003
	(0.004)	(0.012)	(0.011)	(0.010)	(0.011)	(0.012)	(0.013)	(0.011)
Income	0.021^{*}	0.043	-0.017	0.004	0.044^{*}	-0.046	0.022	-0.016
	(0.009)	(0.026)	(0.024)	(0.023)	(0.019)	(0.032)	(0.021)	(0.021)
Age	-0.001^{***}	-0.002^{***}	-0.002^{***}	-0.000	-0.000	0.001^{*}	0.001	-0.000
	(.)							
DK	0.093^{***}							
	(0.008)							
FR	0.003							
	(0.007)							
GB	0.010							
	(0.007)							
IT	0.056^{***}							
	(0.007)							
SK	-0.002							

	(0.007)							
US	-0.055***							
	(0.008)							
Constant	0.018	0.039	0.117**	-0.018	0.011	0.083*	0.142**	0.017
	(0.018)	(0.042)	(0.044)	(0.048)	(0.040)	(0.042)	(0.053)	(0.043)
N	9671	1431	1429	1367	1448	1384	1179	1433

Robust standard errors in parentheses; * $p < 0.05$, ** $p < 0.01$, *** $p < 0.001$

Table B3: Revised model explaining inclusive redistribution with anti-immigrant scale

	All	US	CA	GB	IT	FR	DK	SK
General Redistribution	0.396***	0.424***	0.415***	0.381***	0.396***	0.239***	0.261***	0.422***
	(0.011)	(0.029)	(0.028)	(0.029)	(0.027)	(0.028)	(0.030)	(0.026)
Membership	0.323***	0.338***	0.281***	0.402***	0.254***	0.291***	0.397***	0.273***
	(0.012)	(0.030)	(0.032)	(0.030)	(0.029)	(0.030)	(0.036)	(0.034)
National ID	0.002	-0.066*	-0.010	0.004	0.060*	0.019	0.032	0.017
	(0.009)	(0.027)	(0.024)	(0.022)	(0.023)	(0.026)	(0.031)	(0.022)
Ethnic nat'l	-0.002	0.064***	0.008	0.008	-0.032	-0.035*	-0.002	-0.037*
	(0.007)	(0.019)	(0.018)	(0.019)	(0.017)	(0.018)	(0.023)	(0.017)
Civic nat'l	0.053***	-0.009	0.051	0.057*	0.084**	0.083**	0.067*	0.043
	(0.010)	(0.028)	(0.031)	(0.025)	(0.027)	(0.027)	(0.032)	(0.025)
Immigrants' Control (lack)	-0.002	0.012	-0.032*	0.009	-0.011	-0.009	0.011	-0.017
	(0.007)	(0.019)	(0.016)	(0.017)	(0.018)	(0.019)	(0.023)	(0.017)
Immigrants' Level of Need	0.050***	0.004	0.032	0.047*	0.089***	0.040	0.068*	0.095***
	(0.010)	(0.025)	(0.024)	(0.024)	(0.023)	(0.025)	(0.031)	(0.023)
Anti-Immigrant	-0.319***	-0.313***	-0.301***	-0.321***	-0.293***	-0.403***	-0.337***	-0.319***
	(0.014)	(0.033)	(0.034)	(0.034)	(0.033)	(0.035)	(0.040)	(0.035)
Right-leaning	0.003	0.021	0.020	-0.004	-0.009	-0.002	-0.017	-0.009
	(0.005)	(0.013)	(0.012)	(0.012)	(0.012)	(0.012)	(0.014)	(0.012)
Left-leaning	0.018***	0.020	-0.005	0.026*	0.020	0.010	0.019	0.033*
	(0.005)	(0.017)	(0.012)	(0.013)	(0.011)	(0.013)	(0.015)	(0.013)
Man	-0.011**	0.017	-0.002	-0.014	-0.024**	-0.000	-0.036**	-0.025*
	(0.004)	(0.011)	(0.009)	(0.010)	(0.009)	(0.010)	(0.012)	(0.010)
Christian	0.006	0.029*	-0.012	0.009	-0.008	0.007	-0.001	0.008
	(0.004)	(0.013)	(0.010)	(0.010)	(0.011)	(0.010)	(0.013)	(0.010)
Other religions	0.006	0.032	-0.010	0.005	0.006	0.026	-0.006	-0.004
	(0.008)	(0.019)	(0.015)	(0.018)	(0.053)	(0.020)	(0.021)	(0.019)
Foreign born	0.003	0.012	0.013	0.013	-0.016	0.001	-0.013	-0.006
	(0.007)	(0.024)	(0.012)	(0.017)	(0.028)	(0.022)	(0.026)	(0.015)
University Degree	0.007	0.019	0.010	-0.005	-0.008	0.016	0.023	-0.002
	(0.004)	(0.012)	(0.010)	(0.010)	(0.011)	(0.011)	(0.012)	(0.010)
Income	0.016	0.040	-0.021	-0.001	0.038*	-0.035	0.010	-0.025
	(0.008)	(0.025)	(0.022)	(0.021)	(0.019)	(0.030)	(0.020)	(0.020)
Age	-0.001***	-0.002***	-0.002***	-0.000	0.000	0.001	0.001	-0.000
	(0.000)	(0.000)	(0.000)	(0.000)	(0.000)	(0.000)	(0.000)	(0.000)
DK	0.084***							
	(0.008)							
FR	0.011							
	(0.007)							
GB	0.007							
	(0.007)							
IT	0.072***							
	(0.007)							
SK	0.015*							
	(0.007)							
US	-0.056***							
	(0.007)							

Constant	0.251***	0.254***	0.341***	0.229***	0.238***	0.392***	0.333***	0.273***
	(0.018)	(0.044)	(0.046)	(0.047)	(0.046)	(0.045)	(0.056)	(0.045)
N	10351	1533	1547	1487	1504	1475	1269	1536

Robust standard errors in parentheses; * $p < 0.05$, ** $p < 0.01$, *** $p < 0.001$

Table B4: Pooled model explaining inclusive redistribution with country interaction (Figure 4)

General Redistribution	0.397***
	(0.012)
Membership	0.447***
	(0.024)
DK	0.090***
	(0.019)
FR	0.062***
	(0.018)
GB	0.098***
	(0.016)
IT	0.166***
	(0.016)
SK	0.173***
	(0.018)
US	0.085***
	(0.016)
DK*Membership	-0.072*
	(0.034)
FR*Membership	0.019
	(0.032)
GB*Membership	-0.093**
	(0.032)
IT*Membership	-0.139***
	(0.032)
SK*Membership	-0.047
	(0.036)
US*Membership	-0.068*
	(0.034)
National ID	-0.003
	(0.010)
Ethnic nat'l	-0.038***
	(0.007)
Civic nat'l	0.050***
	(0.011)
Immigrants' Control (Lack)	-0.046***
	(0.007)
Immigrants' Level of Need	0.086***
	(0.010)
Thermometer (Affect)	0.154***
	(0.010)
Right-leaning	-0.012*
	(0.005)
Left-leaning	0.026***
	(0.005)
Man	-0.013***
	(0.004)

Christian	0.000
	(0.004)
Other religions	0.003
	(0.008)
Foreign-born	0.000
	(0.007)
University	0.011**
	(0.004)
Income	0.019*
	(0.009)
Age	-0.001***
	(0.000)
Constant	-0.062**
	(0.020)
N	9671

Robust standard errors in parentheses
* $p < 0.05$, ** $p < 0.01$, *** $p < 0.001$

Appendix C: Institutional and Migration Context by Country

	MIPEX (2020)	*MCP (2020)*	*% Foreign-Born*[1]	*Difference in share of migrants 2010-2020*[2]
SE	86	7	19.5%	5.6
CA	80	7	21.0%	1.5
US	73	3.5	13.6%	3.5
IT	58	1.5	10.4%	0.8
FR	56	1.5	12.8%	1.4
UK	56	6	13.7%	2.6
DK	49	1	10.5%	3.4

[1] Based on most recent data available from the OECD, https://data.oecd.org/migration/foreign-born-population.htm#indicator-chart
[2] Data drawn from the Migration Data Portal, https://www.migrationdataportal.org/ for 2020.
